# Supplementary material for: The potential value of thromboelastography and conventional coagulation detection in guiding the management of venous thrombosis after traumatic brain injury
Source: PLoS One. 2026 Feb 20;21(2):e0343043. doi: 10.1371/journal.pone.0343043 (PMC12923022; doi:10.1371/journal.pone.0343043)
Supplement: Attachment — Submitted filename: Reviewer Commen.docx [file pone.0343043.s002.docx]

**Reviewer Comments**

Zhanfeng Niu et al had done study on the Potential Value of Thromboelastography and Conventional Coagulation Detection in Guiding the Management of Venous Thrombosis after Traumatic Brain Injury. This study addresses a clinically relevant question and provides valuable longitudinal data on coagulation dynamics after isolated traumatic brain injury.

**Comments**

1. **Sample Size and Statistical Power**
   The final analysis includes a relatively small cohort (n = 71), with only 30 thrombosis events. This raises concerns regarding statistical power, particularly for multivariate logistic regression analyses involving multiple covariates. The possibility of model overfitting cannot be excluded, and a formal power calculation is not provided. The authors should acknowledge this limitation more explicitly and interpret regression findings cautiously.
2. **Retrospective, Single-Center Design**
   The retrospective single-center nature of the study introduces potential selection bias and limits external validity. Institutional practices related to thromboprophylaxis, imaging frequency, and ICU management may influence both coagulation parameters and thrombosis rates. This limitation should be emphasized in the Discussion.
3. **Lack of Standardization of Thromboprophylaxis**
   The manuscript does not clearly describe the timing, type, or consistency of pharmacological thromboprophylaxis across patients. Given the strong influence of anticoagulation on both conventional coagulation tests and TEG parameters, this represents a significant confounder. Clarification is required, or this should be addressed as a major limitation.
4. **Potential Detection Bias for DVT**
   DVT diagnosis was based on Doppler ultrasound and pulmonary angiography; however, the frequency and indications for screening are not specified. Patients with more severe TBI or prolonged immobilization may have undergone more frequent imaging, potentially leading to detection bias. This issue should be discussed.
5. **Interpretation of TEG Findings**
   Although TEG parameters demonstrated a persistent hypercoagulable state in the thrombosis group, they did not retain independent predictive value in multivariate analysis. This negative finding may be due to limited sample size rather than true lack of association. The conclusion regarding the limited predictive utility of TEG should therefore be tempered.
6. **Incomplete Adjustment for Confounders**
   Several known risk factors for venous thrombosis—including duration of immobilization, mechanical ventilation, central venous catheterization, transfusion requirements, and infection—were not included in regression models. Residual confounding may influence the reported associations.
7. **Causality Cannot Be Established**
   Given the observational design, causal relationships between coagulation parameters and thrombosis cannot be inferred. The Discussion occasionally implies mechanistic interpretation, which should be clearly framed as speculative.
8. **Clarification of TEG Methodology**
   Only kaolin-activated TEG was used. The authors should acknowledge that this approach may not fully capture fibrinolytic activity or platelet inhibition pathways.
9. **Timing of Coagulation Changes**
   The predefined time points (24, 72, and 168 hours) may miss early or transient coagulation changes immediately post-injury. This limitation should be briefly mentioned.
10. **Language and Grammar**
    Minor grammatical inconsistencies and typographical errors are present throughout the manuscript and should be corrected during revision.
11. **Figures and Tables**
    Some tables are dense and may benefit from simplification or clearer labeling to improve readability.
