# Supplementary material for: The potential value of thromboelastography and conventional coagulation detection in guiding the management of venous thrombosis after traumatic brain injury
Source: PLoS One. 2026 Feb 20;21(2):e0343043. doi: 10.1371/journal.pone.0343043 (PMC12923022; doi:10.1371/journal.pone.0343043)
Supplement: Attachment — Submitted filename: Response to Reviewers.docx [file pone.0343043.s005.docx]

**Manuscript ID:** PONE-D-25-62755

**Title:** The Potential Value of Thromboelastography and Conventional Coagulation Detection in Guiding the Management of Venous Thrombosis after Traumatic Brain Injury

Dear Editors and Reviewers,

Thank you for your letter and for the reviewer’s comments concerning our manuscript entitled “The Potential Value of Thromboelastography and Conventional Coagulation Detection in Guiding the Management of Venous Thrombosis after Traumatic Brain Injury” (PONE-D-25-62755). Those comments are all valuable and very helpful for revising and improving our manuscript, as well as the important guiding significance to our researches. We have studied comments carefully and have made correction which we hope will meet with your approval. Revised portion are marked in red in the manuscript. Furthermore, the point-to-point responses have also been presented in this response letter.

We are grateful to you for allowing us to revise our manuscript and look forward to hearing from you soon.

Best wishes

Sincerely,

Zhanfeng Niu

**Response to Academic Editor and Journal Requirements**

**Comment 1:** PLOS ONE style and file naming requirements

**Response:** We have carefully revised the manuscript to comply with PLOS ONE formatting and style requirements in accordance with the provided templates. Specifically, we changed the format of the title to be written in sentence case. And all the main sections have been given first-level headings.

**Comment 2:** Inconsistency in grant information

**Response:** Thank you for pointing this out. We have reviewed the information provided in the ‘Funding Information’ and ‘Financial Disclosure’ sections and have ensured that they now match and are consistent with each other.

**Comment 3:** Funding information incorrectly included in the Acknowledgments section

**Response:** We thank the editor for this clarification. All funding-related text has been removed from the Acknowledgments section of the manuscript. Funding information is now reported exclusively in the Funding Statement via the online submission form. We have included the amended Funding Statement in the cover letter and kindly request that the submission system be updated accordingly.

**Comment 4:** Ethics statement location

**Response:** We have revised the manuscript so that the ethics statement appears only in the Methods section, and any duplicate statements in other sections have been removed.

**Comment 4:** **Missing in-text reference to Table 5**

**Response:** Thank you for noting this. We have now added an explicit in-text reference to Table 5 in the Results section to ensure proper linkage. (page 17, line 295)

**Comment 6: Suggested citations**

**Response:** We confirm that the reviewer comments did not include specific recommendations to cite additional previously published works. Therefore, no changes were required in this regard.

**Responds to the reviewer’s comments:**

**Reviewer #1:**

1. The rationale for selecting the study period should be clarified, particularly the non-standard end date of February 2.

**Response:** We appreciate the reviewer's point. The study period endpoint (February 2, 2022) was determined by the need to maintain consistency in core measurement data. This study focuses on the comparison of detailed coagulation parameters, including thromboelastography (TEG) indices, across different time points and patient groups. The TEG equipment used in our institution underwent a scheduled upgrade in February 2022, which could have affected absolute values and reference ranges. To avoid potential bias related to differences in measurement systems and to ensure comparability of coagulation data across patients, only individuals who completed all required testing prior to the equipment upgrade were included in the analysis. As a result, February 2, 2022 represents the natural endpoint of the study period.

2. The rationale for including sleep disorders and mental disorders among the exclusion criteria is unclear.

**Response:** Thank you to the reviewers for pointing this out. Regarding the exclusion of patients with mental disorders, this decision was primarily based on careful consideration of controlling for confounding medication factors and ensuring data reliability. For patients with mental disorders, neither the patients themselves nor their family members could accurately provide a complete history of long-term oral medications that might affect coagulation function. Therefore, to ensure the quality of the data upon which our analysis relies and to avoid drawing biased conclusions due to uncontrolled confounding medication factors, we decided to exclude these patients from the study cohort.

The exclusion of patients with sleep disorders, such as those with obstructive sleep apnea (OSA), is supported by substantial evidence demonstrating that OSA is an independent risk factor for deep vein thrombosis and pulmonary embolism^[1, 2]^. Including such patients would prevent us from clearly discerning to what extent the observed abnormalities in coagulation parameters and thrombotic events in our study could be attributed to acute TBI itself, leading to this exclusion decision.

[1]	PENG Y-H, LIAO W-C, CHUNG W-S, et al. Association between obstructive sleep apnea and deep vein thrombosis / pulmonary embolism: A population-based retrospective cohort study [J]. Thrombosis Research, 2014, 134(2): 340-5.

[2]	ZHANG J, GU J, KUANG Y, et al. Prevalence of obstructive sleep apnea in venous thromboembolism: a systematic review and meta-analysis [J]. Sleep and Breathing, 2019, 23(4): 1283-9.

3. Excluding patients who received anticoagulants affecting the coagulation system within 7 days post-injury may have excluded cases in which venous thrombosis developed early, and anticoagulation was initiated during that period. If so, this constitutes an important limitation that should be explicitly acknowledged. In addition, it should be made clear that the results and discussion primarily concern venous thrombosis confirmed after 7 days post-trauma.

**Response:** We thank the reviewer for raising this important concern. The exclusion criterion stating “use of medications that may affect coagulation function within 7 days post-injury” was intended to exclude patients receiving chronic antiplatelet or anticoagulant therapy (such as aspirin or clopidogrel) for pre-existing cardiovascular diseases.

The timing of pharmacological anticoagulation in patients with TBI varies considerably in clinical practice, ranging from as early as 72 hours to several weeks after injury. However, considering the risk of hemorrhagic complications, in our institution, pharmacological anticoagulation is usually initiated no earlier than 7 days after injury and only after radiological confirmation of intracranial hemorrhage stability. Therefore, initiation of anticoagulation within the first 7 days after injury is uncommon in our center.

The primary objective of this study was to characterize the natural evolution of coagulation profiles and the risk of thrombosis in patients with isolated TBI who did not receive early pharmacological anticoagulation. Initiation of oral or systemic anticoagulant therapy within 7 days post-injury would inevitably interfere with the spontaneous process of thrombus formation, rendering such patients unrepresentative of an untreated TBI-associated thrombosis risk population.

Furthermore, all venous thrombotic events included in this study were diagnosed after day 7 post-injury using lower-extremity Doppler ultrasound or computed tomography pulmonary angiography (CTPA). We have now clarified this point in the Methods section and emphasized the importance of this time window for interpretation of our findings in the Discussion.

To address this comment and improve methodological clarity, we have made the following revisions to the manuscript:

a. **Methods – Patient population** (page 6, lines 119-122)

We have clarified the definition of anticoagulant exposure within 7 days post-injury.

Added text:

The exclusion criterion regarding the use of medications affecting coagulation function within 7 days post-injury specifically referred to chronic antiplatelet or anticoagulant therapy prescribed for pre-existing cardiovascular diseases (e.g., aspirin or clopidogrel).

b. **Methods – Outcomes** (page 8, lines 167-168)

We have explicitly reported the timing and diagnostic modalities of venous thrombosis.

Added text:

All venous thrombotic events included in this study occurred more than 7 days after injury.

c. **Discussion** (page 21, lines 358-360)

We have emphasized the study population and the interpretation of findings in relation to the predefined time window.

Added text:

By focusing on patients who did not receive early pharmacological anticoagulation and on thrombosis diagnosed after day 7 post-injury, this study specifically reflects the natural coagulation evolution and untreated thrombosis risk in isolated TBI patients.

4. Because coagulation markers can vary substantially with time after trauma, information on the interval from injury to hospital presentation should be provided.

**Response:** We appreciate the reviewers' suggestions. This study focuses on systemic coagulation changes at predefined time points of approximately 24, 72, and 168 hours post-injury, rather than the transient fluctuations in the extremely early stage (< 24 hours). All patients were tested at the 24-hour time point after injury, ensuring the consistency of the time points. Although there were differences in the specific time from injury to admission, the focus of this study is on the longitudinal coagulation trend within one week after injury, rather than the absolute values at the moment of admission. Therefore, we believe that the current design can effectively capture the evolution pattern of thrombosis risk related to clinical practice and provide a basis for early warning.

5. The definition of the primary outcome is unclear. The authors should specify how venous thrombosis was diagnosed, including the diagnostic modality and whether imaging was performed systematically or only when clinically suspected. If a standardized surveillance protocol was used, it should be described.

**Response:** We thank the reviewers for pointing out this key issue. In this study, venous thrombosis was defined as deep vein thrombosis (DVT) and/or pulmonary embolism confirmed by objective imaging modalities. DVT was diagnosed using lower-extremity Doppler ultrasound, and pulmonary embolism was confirmed by computed tomography pulmonary angiography.

Imaging examinations were not performed as part of a routine systematic screening protocol for all patients. Instead, Doppler ultrasound and/or computed tomography pulmonary angiography were performed based on comprehensive clinical judgment, including limb swelling, unexplained hypoxemia, sudden deterioration in oxygenation, or elevated thrombotic risk as judged by the treating physicians.

To improve clarity, we have revised the Methods section to explicitly define the primary outcome and describe the diagnostic modalities and indications for imaging. To improve clarity and avoid redundancy, we have revised the Methods section by consolidating all information related to outcome definition, diagnostic modalities, and imaging indications into the Outcomes subsection. Accordingly, the original sentence describing thrombus detection has been removed, as these details are now explicitly and comprehensively defined in the Outcomes section.

**Methods – Outcomes** (page 8, lines 162-167)

Added text:

The primary outcome of this study was venous thrombosis, defined as the occurrence of DVT and/or pulmonary embolism. DVT was diagnosed using lower-extremity Doppler ultrasound, and pulmonary embolism was confirmed by computed tomography pulmonary angiography. Imaging examinations were performed based on the comprehensive clinical judgment of the attending physician rather than as part of a routine systematic screening protocol.

6. For patients in the thrombosis group, the timing of thrombosis diagnosis should be reported. Interpretation differs significantly depending on whether laboratory/TEG values were obtained before or after thrombosis onset.

**Response:** We thank the reviewer for this important comment. In the present study, all venous thrombotic events were diagnosed after day 7 post-injury, using lower-extremity Doppler ultrasound and/or computed tomography pulmonary angiography. Importantly, all laboratory coagulation parameters and TEG measurements at 24, 72, and 168 hours were obtained prior to thrombosis diagnosis.

Therefore, the coagulation and TEG values analyzed in this study reflect coagulation changes preceding thrombosis detection, rather than alterations secondary to the thrombotic event itself or to anticoagulant therapy initiated after diagnosis. This temporal relationship supports the interpretation of these parameters as potential early indicators of thrombosis risk.

To improve clarity, we removed a previously existing sentence describing coagulation and TEG measurements at 24, 72, and 168 hours post-injury and we have explicitly reported the timing of thrombosis diagnosis and its relationship to laboratory and TEG measurements in the Methods section.

**Methods –Data collection and definitions** (page 8, lines 158-160)

Added text:

Coagulation parameters and TEG measurements at 24, 72, and 168 hours were obtained prior to thrombosis diagnosis in all patients.

**Methods – Outcomes** (page 8, lines 167-168)

Added text:

All venous thrombotic events included in this study occurred more than 7 days after injury.

7. Given the availability of clinical practice guidelines for the prevention of venous thromboembolism, it is necessary to describe the specific preventive measures implemented in this study.

**Response:** We thank the reviewer for this important comment. During the study period, venous thromboembolism prevention in patients with isolated traumatic brain injury primarily consisted of mechanical prophylaxis, including intermittent pneumatic compression devices and early mobilization when clinically feasible. Pharmacological thromboprophylaxis was generally not initiated during the first 7 days after injury, due to concerns regarding hemorrhagic progression, and was considered only after radiological confirmation of intracranial hemorrhage stability.

Thus, the study cohort largely represents isolated TBI patients managed without early pharmacological anticoagulation, allowing assessment of coagulation dynamics and thrombosis risk in the early post-injury phase.

To improve clarity and transparency, we have added a description of VTE preventive measures to the Methods section.

**Methods – Peri-injury management** (page 9, lines 170-174)

Added text:

During the study period, venous thromboembolism prevention primarily consisted of mechanical prophylaxis, including intermittent pneumatic compression devices and early mobilization when clinically feasible. Pharmacological thromboprophylaxis was generally not administered within the first 7 days after injury and was considered only after radiological confirmation of intracranial hemorrhage stability.

8. For Tables 1–3, the 95% confidence intervals should be reported in addition to the p-values.

**Response:** Thank you for this valuable suggestion. We have added 95% confidence intervals (95% CI) for all between-group comparisons in Tables 2 and 3. No 95% CI was added to Table 1, as it presents baseline characteristics without inferential comparisons.

9. For “The coagulation status was categorized into normal, hypercoagulable, and hypocoagulable states.” (Page 12, lines 211-213), definitions for these three categories should be provided in the Methods section.

**Response:** We thank the reviewer for this important comment. We have now explicitly defined the criteria used to classify coagulation status as normal, hypercoagulable, or hypocoagulable based on standard TEG reference ranges.

**Methods – Data collection and definitions** (page 8, lines 153-158)

Added text:

Based on standard TEG reference ranges, coagulation status was categorized as follows: a hypercoagulable state was defined by shortened R time and/or elevated α-angle or MA above the upper reference limits; a hypocoagulable state was defined by prolonged R time and/or reduced α-angle or MA below the lower reference limits; patients not meeting these criteria were classified as having normal coagulation status.

10. Since K-time is described in the Methods section, corresponding results for K-time should also be presented, or the reason for omission should be stated.

**Response:** We thank the reviewer for raising this point. K time was measured and recorded as part of the standard thromboelastography assessment in this study. However, K time primarily reflects the kinetics of fibrin clot formation and is highly correlated with the α angle. In our dataset, K time demonstrated a strong inverse correlation with the α angle, indicating substantial collinearity between these parameters.

To avoid redundancy and multicollinearity and to improve the stability of the multivariable models, the α angle was selected to represent clot formation dynamics in the final analyses. K time was therefore not included as an independent variable.

In the revised manuscript, we have addressed this point by adding an explicit explanation in the Statistical analysis subsection of the Methods section.

**Methods – Statistical analysis** (page 9, lines 189-192)

Added text:

Given the high collinearity observed between the TEG parameters K-time and α-angle, which would compromise model stability, we selected the α-angle as the representative parameter for clot kinetics in the multivariate analysis, and K-time was not included as an independent variable.

11. With this sample size and number of outcome events, including seven predictors in a multivariable logistic regression model may exceed commonly recommended events-per-variable thresholds.

**Response:** We thank the reviewer for this important methodological comment. We agree that, given the single-center, retrospective design and limited sample size, the robustness of the multivariable logistic regression analyses may be constrained, including an increased risk of overfitting.

To address this concern, we have revised the Limitations section of the manuscript to more explicitly acknowledge the implications of the limited sample size for the stability and interpretation of the multivariable analyses. The revised text emphasizes that these constraints may affect the generalizability of the findings and necessitate cautious interpretation of the regression results.

In light of these considerations, the multivariable analyses are interpreted cautiously and are not intended to serve as definitive predictive models, but rather to provide insight into potential associations that merit further investigation.

**Limitation** (page 23, lines 414-416)

Revised text:

First, the single-center, retrospective design with a limited sample size may limit generalizability, preclude severity-stratified analyses, and increase the risk of overfitting.

12. The Methods section does not describe the methods used for correlation analysis. Furthermore, it is unclear whether this was performed in all patients without stratification.

**Response:** We thank the reviewer for this helpful comment. In this study, correlation analyses were performed to assess the associations between coagulation parameters, thromboelastography (TEG) variables, and thrombosis-related outcomes. Given that most laboratory and TEG variables did not follow a normal distribution, Spearman’s rank correlation analysis was used.

The correlation analyses were conducted in the overall study population without stratification, as the primary aim was to evaluate the general relationships between coagulation-related variables and thrombosis risk across the entire cohort. No subgroup- or stratification-based correlation analyses were performed.

We have now clarified the statistical methods used for correlation analysis and the study population in which these analyses were conducted in the Methods section.

**Methods – Statistical analysis** (page 9, lines 184-187)

Added text:

Correlation analyses were performed using Spearman’s rank correlation coefficient to evaluate the associations between coagulation parameters, thromboelastography variables, and thrombosis-related outcomes. These analyses were conducted in the overall study population without stratification.

13. Table 5 reports p-values only as thresholds (e.g., p > 0.05) rather than as exact values.

**Response:** Thank you for your careful review. We have revised Table 5 to improve the accuracy and clarity of statistical reporting. As part of this revision, a transcription error in the original Table 5B, in which an incorrect “<” symbol was reported for one p-value, has been corrected, and the table has been updated accordingly.

These corrections do not affect the interpretation of the results. The corresponding descriptions in the Results section have been checked to ensure consistency with the revised Table 5.

14. "In our cohort, the thrombosis group exhibited significantly elevated PT and D-D levels compared to the non-thrombosis group. This pattern suggests substantial consumption of coagulation factors and rapid fibrin formation and turnover, consistent with systemic activation of the coagulation cascade following brain injury." (Page 20, lines 306-309) Given this interpretation, which may suggest a DIC-like pathophysiology, the finding of elevated fibrinogen appears atypical. Further discussion is needed to address this apparent inconsistency.

**Response:** We thank the reviewer for this insightful comment regarding the apparent inconsistency between elevated PT and D-dimer levels and increased fibrinogen concentrations.

Although prolonged PT and elevated D-dimer may resemble features observed in disseminated intravascular coagulation (DIC), the coagulation abnormalities following traumatic brain injury represent a distinct pathophysiological process rather than classic consumptive DIC. In the early and subacute phases after TBI, fibrinogen often behaves as an acute-phase reactant, and its synthesis can be upregulated in response to systemic inflammation and tissue injury. Consequently, elevated fibrinogen levels may coexist with markers of coagulation activation and fibrin turnover, such as increased D-dimer.

Moreover, TBI-associated coagulopathy is increasingly recognized as a dynamic and heterogeneous process, characterized by simultaneous activation of coagulation and inflammatory pathways, rather than uniform depletion of coagulation factors. Therefore, the observed elevation in fibrinogen in our thrombosis group does not contradict the evidence of coagulation cascade activation but instead reflects a hypercoagulable and prothrombotic state distinct from classical DIC.

We have revised the Discussion section to explicitly address this point and clarify the pathophysiological interpretation of these findings.

**Discussion** (page 20, lines 336-346)

Added text:

However, given the observational nature of this study, these findings should be interpreted as descriptive associations rather than evidence of a causal or mechanistic relationship. The proposed pathophysiological interpretations remain speculative. Although prolonged PT and elevated D-D levels may resemble features of disseminated intravascular coagulation, TBI-associated coagulopathy represents a distinct and dynamic pathophysiological process. In the early and subacute phases following TBI, fibrinogen may increase as an acute-phase reactant in response to systemic inflammation and tissue injury. Thus, elevated fibrinogen levels can coexist with markers of coagulation activation and fibrin turnover, reflecting a hypercoagulable and prothrombotic state rather than classical consumptive disseminated intravascular coagulation.

15. The conclusion states that PT and D-dimer are practical and effective tools for predicting venous thrombosis risk. However, the between-group difference in PT appears to be approximately 1 second, with both values close to the normal range. The clinical application of these findings should be clarified.

**Response:** We thank the reviewer for this important and clinically relevant comment. We agree that although the difference in PT between the thrombosis and non-thrombosis groups reached statistical significance, the absolute difference was relatively small (approximately 1 second) and remained close to the normal reference range. Therefore, PT should not be interpreted in isolation as a diagnostic or decision-making marker for venous thrombosis.

In this study, prolonged PT may reflect subtle but persistent activation of the coagulation system rather than a clinically decisive threshold. When interpreted in combination with elevated D-dimer levels, TEG findings, and clinical risk factors such as injury severity and immobilization, these modest PT changes may still contribute to thrombosis risk stratification.

To avoid overstatement, we have revised the Conclusion section to clarify that PT and D-dimer should be regarded as adjunctive indicators within a multimodal assessment framework, rather than as independent or definitive predictors of venous thrombosis.

**Conclusions** (page 24, lines 429-439)

Revised text

In patients with isolated TBI, dynamic changes in conventional coagulation parameters and TEG within the first week after injury reflect a sustained hypercoagulable state that is may be associated with subsequent venous thrombosis. Patients who developed thrombosis exhibited persistently elevated D-D levels and modest prolongation of prothrombin time across multiple time points, indicating ongoing activation of the coagulation system during the post-injury period. Although TEG parameters consistently suggested hypercoagulability in the thrombosis group, they did not demonstrate independent predictive value in multivariable analyses, which may be attributable to the limited sample size and number of outcome events. Accordingly, TEG findings should be interpreted as reflecting global coagulation tendencies rather than serving as standalone predictors.

16. The reference list appears to require substantial revision.

**Response:** We thank the reviewer for this comment. We have carefully reviewed and revised the entire reference list to ensure accuracy, consistency, and compliance with the journal’s formatting requirements.

**Reviewer #2:**

1. When was the DVT diagnosis made? Wouldn't the timing of thrombus formation affect coagulation parameters differently? This factor was not included.

**R****esponse:** We thank the reviewer for this important comment. As clarified in the revised manuscript, all venous thrombotic events were diagnosed after day 7 post-injury using lower-extremity Doppler ultrasound and/or computed tomography pulmonary angiography. Importantly, all conventional coagulation parameters and TEG measurements (at 24, 72, and 168 hours) were obtained prior to thrombosis diagnosis in all patients.

Therefore, the laboratory and TEG data analyzed in this study reflect coagulation changes preceding thrombosis detection, rather than alterations secondary to established thrombosis or anticoagulant treatment.

This temporal framework has now been explicitly described in the Methods section and considered in the interpretation of the results.

**Methods –Data collection and definitions** (page 8, lines 158-160)

Added text:

Coagulation parameters and TEG measurements at 24, 72, and 168 hours were obtained prior to thrombosis diagnosis in all patients.

**Methods – Outcomes** (page 8, lines 162-168)

Added text:

The primary outcome of this study was venous thrombosis, defined as the occurrence of DVT and/or pulmonary embolism. DVT was diagnosed using lower-extremity Doppler ultrasound, and pulmonary embolism was confirmed by computed tomography pulmonary angiography. Imaging examinations were performed based on the comprehensive clinical judgment of the attending physician rather than as part of a routine systematic screening protocol. All venous thrombotic events included in this study occurred more than 7 days after injury.

2. Pathological conditions vary considerably depending on the severity of head trauma. Perhaps stratified analysis taking this into account is necessary?

**Response:** We thank the reviewer for this important suggestion.

We agree that injury severity may influence coagulation abnormalities after traumatic brain injury. In the present study, head injury severity was incorporated into the analysis as a covariate (including Glasgow Coma Scale score and radiological severity indicators), and its potential confounding effect was therefore partially accounted for in the multivariable models.

However, further stratified analyses based on injury severity were not performed due to the relatively limited sample size and the risk of reduced statistical power within subgroups. Importantly, the primary objective of this study was to characterize overall coagulation trends and thrombosis risk in isolated TBI patients, rather than to establish severity-specific prediction models.

This consideration has now been acknowledged in the Discussion as a limitation, and future large-scale studies will be required to explore severity-stratified coagulation patterns in greater detail.

**Discussion – Limitations** (page 8, lines 415-417)

Revised text:

First, the single-center, retrospective design with a limited sample size may limit generalizability, preclude severity-stratified analyses, and increase the risk of overfitting.

**Reviewer #3:**

1. Sample Size and Statistical Power
The final analysis includes a relatively small cohort (n = 71), with only 30 thrombosis events. This raises concerns regarding statistical power, particularly for multivariate logistic regression analyses involving multiple covariates. The possibility of model overfitting cannot be excluded, and a formal power calculation is not provided. The authors should acknowledge this limitation more explicitly and interpret regression findings cautiously.

**Response:** We thank the reviewer for this insightful methodological comment. We fully acknowledge that the relatively small sample size and limited number of thrombosis events may restrict statistical power and increase the risk of model overfitting in multivariable logistic regression analyses.

To address this concern, we have revised the Limitations section of the manuscript to more explicitly acknowledge the implications of the limited sample size for the stability and interpretation of the multivariable analyses. The revised text emphasizes that these constraints may affect the generalizability of the findings and necessitate cautious interpretation of the regression results.

In light of these considerations, the multivariable analyses are interpreted cautiously and are not intended to serve as definitive predictive models, but rather to provide insight into potential associations that merit further investigation.

**Discussion – Limitations** (page 8, lines 415-417)

Added text:

First, the single-center, retrospective design with a limited sample size may limit generalizability, preclude severity-stratified analyses, and increase the risk of overfitting.

2. Retrospective, Single-Center Design
The retrospective single-center nature of the study introduces potential selection bias and limits external validity. Institutional practices related to thromboprophylaxis, imaging frequency, and ICU management may influence both coagulation parameters and thrombosis rates. This limitation should be emphasized in the Discussion.

**Response:** We thank the reviewer for highlighting this important limitation.

We agree that the retrospective, single-center design may introduce selection bias and limit the external validity of our findings. In particular, institution-specific practices related to thromboprophylaxis strategies, imaging indications, and ICU management may influence coagulation parameters as well as thrombosis detection rates.

To address this concern, we have strengthened the Limitations subsection of the Discussion by explicitly acknowledging the potential impact of center-specific clinical practices on coagulation profiles and venous thrombosis outcomes. We have also emphasized the need for future large-scale, prospective, multicenter studies to confirm the generalizability of our findings.

**Discussion – Limitations**

Revised text:

Additionally, institutional practices regarding thromboprophylaxis and imaging indications may have influenced thrombosis detection, and the use of clinically indicated rather than systematic imaging may have introduced detection bias. (page 23, lines 418-420)

Future large-scale, prospective, multicenter studies are warranted to validate these findings and to further clarify the clinical role of TEG and conventional coagulation parameters in thrombosis risk assessment after TBI. (page 24, lines 424-427)

3. Lack of Standardization of Thromboprophylaxis
The manuscript does not clearly describe the timing, type, or consistency of pharmacological thromboprophylaxis across patients. Given the strong influence of anticoagulation on both conventional coagulation tests and TEG parameters, this represents a significant confounder. Clarification is required, or this should be addressed as a major limitation.

**Response:** We thank the reviewer for this important comment. This issue has been clarified in the revised manuscript. We now explicitly state that pharmacological anticoagulation was generally initiated no earlier than day 7 post-injury in our institution and only after radiological confirmation of intracranial hemorrhage stability. As a result, all conventional coagulation tests and TEG parameters analyzed at 24, 72, and 168 hours were obtained prior to the initiation of pharmacological thromboprophylaxis.

Therefore, the coagulation and TEG measurements in this study reflect the natural coagulation dynamics before anticoagulant exposure, minimizing the confounding effect of pharmacological thromboprophylaxis on the analyzed parameters.

**Methods - Peri-injury management** (page 9, lines 170-174)

Added text:

During the study period, venous thromboembolism prevention primarily consisted of mechanical prophylaxis, including intermittent pneumatic compression devices and early mobilization when clinically feasible. Pharmacological thromboprophylaxis was generally not administered within the first 7 days after injury and was considered only after radiological confirmation of intracranial hemorrhage stability.

4. Potential Detection Bias for DVT
DVT diagnosis was based on Doppler ultrasound and pulmonary angiography; however, the frequency and indications for screening are not specified. Patients with more severe TBI or prolonged immobilization may have undergone more frequent imaging, potentially leading to detection bias. This issue should be discussed.

**Response:** We thank the reviewer for this important comment. As clarified in the revised manuscript, venous thrombosis was diagnosed using clinically indicated Doppler ultrasound and/or computed tomography pulmonary angiography rather than a standardized surveillance protocol. Consequently, the frequency and indications for imaging were not uniform across patients.

We acknowledge that patients with more severe traumatic brain injury or prolonged immobilization may have undergone more frequent imaging, which could increase the likelihood of thrombosis detection and introduce potential detection bias. This limitation has been explicitly addressed in the Discussion – Limitations section of the revised manuscript.

**Discussion – Limitations** (page 23, lines 418-420)

Added text:

Additionally, institutional practices regarding thromboprophylaxis and imaging indications may have influenced thrombosis detection, and the use of clinically indicated rather than systematic imaging may have introduced detection bias.

5. Interpretation of TEG Findings
Although TEG parameters demonstrated a persistent hypercoagulable state in the thrombosis group, they did not retain independent predictive value in multivariate analysis. This negative finding may be due to limited sample size rather than true lack of association. The conclusion regarding the limited predictive utility of TEG should therefore be tempered.

**Response:** We thank the reviewer for this insightful comment regarding the interpretation of the TEG findings. We agree that the lack of independent predictive value of TEG parameters in the multivariable analyses does not necessarily indicate a true absence of association, and may in part be attributable to the limited sample size and number of outcome events in this study.

In response to this comment, we have tempered the conclusions regarding the predictive utility of TEG. Specifically, we now emphasize that although TEG parameters consistently suggested a hypercoagulable state in patients who developed thrombosis, they did not demonstrate clear independent predictive value in multivariable analyses under the constraints of the current dataset. Accordingly, TEG findings are interpreted as reflecting global coagulation tendencies rather than serving as standalone predictors.

These revisions have been incorporated into the Discussion and Conclusion sections of the revised manuscript.

**Discussion** (page 21, lines 370-377)

Added text:

This finding should be interpreted with caution, as the lack of independent significance may reflect limitations in predictive modeling rather than negating the biological relevance of TEG findings. In the context of a limited sample size and number of outcome events, statistical power may be insufficient to disentangle the contributions of correlated coagulation variables, thereby attenuating their apparent effects in adjusted models. Consequently, while TEG parameters may not function as independent predictors in this analysis, they remain informative for characterizing global coagulation tendencies in patients with isolated TBI.

**Conclusion** (page 24, lines 433-4387)

Revised text:

Although TEG parameters consistently suggested hypercoagulability in the thrombosis group, they did not demonstrate independent predictive value in multivariable analyses, which may be attributable to the limited sample size and number of outcome events. Accordingly, TEG findings should be interpreted as reflecting global coagulation tendencies rather than serving as standalone predictors.

6. Incomplete Adjustment for Confounders
Several known risk factors for venous thrombosis—including duration of immobilization, mechanical ventilation, central venous catheterization, transfusion requirements, and infection—were not included in regression models. Residual confounding may influence the reported associations.

**Response:** We thank the reviewer for this important comment regarding potential residual confounding. This issue has been addressed in the Discussion by framing venous thrombosis after severe traumatic brain injury as a multifactorial clinical syndrome. Specifically, we emphasize that thrombotic risk reflects the combined impact of injury severity, prolonged immobilization, and critical care–related interventions, rather than the effect of any single laboratory or coagulation-related parameter. This perspective is intended to guide appropriate interpretation of the observed associations while acknowledging the complex clinical determinants of thrombosis risk in this population.

**Discussion** (page19, lines 326-330)

Added text:

Accordingly, thrombotic risk following severe TBI reflects the combined impact of trauma severity, prolonged immobilization, and critical care–related interventions. Together, these interacting processes indicate that venous thrombosis in this population represents a multifactorial clinical syndrome rather than the consequence of any single determinant.

7. Causality Cannot Be Established
Given the observational design, causal relationships between coagulation parameters and thrombosis cannot be inferred. The Discussion occasionally implies mechanistic interpretation, which should be clearly framed as speculative.

**Response:** We thank the reviewer for this important clarification. We fully agree that, given the observational and retrospective design of the study, causal relationships between coagulation parameters and venous thrombosis cannot be established. In response to this concern, we have carefully revised the Discussion to ensure that all mechanistic interpretations are clearly framed as speculative hypotheses rather than causal inferences.

**Discussion** (page20, lines 336-339)

Added text:

However, given the observational nature of this study, these findings should be interpreted as descriptive associations rather than evidence of a causal or mechanistic relationship. The proposed pathophysiological interpretations remain speculative.

8. Clarification of TEG Methodology
Only kaolin-activated TEG was used. The authors should acknowledge that this approach may not fully capture fibrinolytic activity or platelet inhibition pathways.

**Response:** We thank the reviewer for this important methodological comment. We agree that kaolin-activated thromboelastography does not fully capture fibrinolytic activity or platelet inhibition pathways. In response, we have revised the Discussion to clarify the interpretative scope of TEG in the present study. Specifically, we now state that kaolin-activated TEG primarily reflects clot initiation and strength and provides an integrated assessment of global coagulation dynamics. Accordingly, TEG findings are interpreted as indicators of overall prothrombotic tendency rather than as a comprehensive evaluation of all hemostatic mechanisms. We believe this clarification appropriately contextualizes the role of TEG in our analysis without overstating its predictive scope.

**Discussion** (page22, lines 377-382)

Added text:

It should be noted that the use of kaolin-activated thromboelastography primarily reflects clot initiation and strength, providing an integrated assessment of global coagulation dynamics. As such, TEG findings in this study are best interpreted as indicators of overall prothrombotic tendency rather than as a comprehensive evaluation of fibrinolytic activity or platelet inhibition pathways.

9. Timing of Coagulation Changes
The predefined time points (24, 72, and 168 hours) may miss early or transient coagulation changes immediately post-injury. This limitation should be briefly mentioned.

**Response:** We thank the reviewer for this helpful comment. We agree that coagulation abnormalities following traumatic brain injury may occur very early after injury and that predefined sampling at 24, 72, and 168 hours may not fully capture very early or transient post-injury changes. This issue has been acknowledged in the manuscript. Specifically, in the Discussion, we now clarify that the precise onset and peak of coagulation dysfunction could not be determined in part because coagulation parameters were assessed at predefined time points, which constrains interpretation of the exact temporal evolution of coagulation abnormalities.

**Discussion → Limitations** (page22, lines 419-423)

Revised text:

Finally, the precise onset and peak of coagulation dysfunction following TBI could not be determined, in part because coagulation parameters were assessed at predefined time points (24, 72, and 168 hours), which constrains interpretation of the exact temporal evolution of coagulation abnormalities.

10. Language and Grammar
Minor grammatical inconsistencies and typographical errors are present throughout the manuscript and should be corrected during revision.

**Response:** We thank the reviewer for this comment. A careful language revision has been performed throughout the manuscript to correct minor grammatical inconsistencies and typographical errors. Representative revisions include correction of subject–verb agreement, standardization of statistical formatting. These changes do not affect the scientific content of the manuscript.

11. Figures and Tables
Some tables are dense and may benefit from simplification or clearer labeling to improve readability.
**Response:** Thank you for this helpful suggestion. We have carefully reviewed all tables and figures to improve clarity and readability. Table titles, column headings, and footnotes have been refined to provide clearer explanations of variables and abbreviations. Where appropriate, formatting adjustments were made to enhance readability while preserving the completeness of the data.
