# Supplementary material for: The potential value of thromboelastography and conventional coagulation detection in guiding the management of venous thrombosis after traumatic brain injury
Source: PLoS One. 2026 Feb 20;21(2):e0343043. doi: 10.1371/journal.pone.0343043 (PMC12923022; doi:10.1371/journal.pone.0343043)
Supplement: Attachment — Submitted filename: comments.docx [file pone.0343043.s004.docx]

The authors have **responded thoroughly and professionally**. Most issues are **appropriately acknowledged**, and limitations are **clearly integrated** into the manuscript. The tone is measured, and overinterpretation has largely been corrected. That said, several responses rely heavily on post-hoc framing rather than methodological mitigation, which should be noted.

The authors have **substantively engaged with my critiques**, strengthened the manuscript’s methodological transparency, and appropriately tempered interpretation. Remaining limitations are largely intrinsic to the study design and are now clearly acknowledged. No major unresolved issues remain.
